# Novel pathogenic *UQCRC2* variants in a female with normal neurodevelopment

**DOI:** 10.1101/mcs.a006295

**Published:** 2023-12

**Authors:** Lea Abou Haidar, Robert C. Harris, Panayotis Pachnis, Hongli Chen, Garrett K. Gotway, Min Ni, Ralph J. DeBerardinis

**Affiliations:** 1 Children's Medical Center Research Institute, UT Southwestern Medical Center, Dallas, Texas 75390, USA;; 2 Howard Hughes Medical Institute, UT Southwestern Medical Center, Dallas, Texas 75390, USA;; 3 Department of Pediatrics, UT Southwestern Medical Center, Dallas, Texas 75390, USA;; 4 Eugene McDermott Center for Human Growth and Development, UT Southwestern Medical Center, Dallas, Texas 75390, USA;; 5 Department of Internal Medicine, UT Southwestern Medical Center, Dallas, Texas 75390, USA

**Keywords:** acute hyperammonemia, decreased activity of mitochondrial complex III, fasting hypoglycemia, stress/infection-induced lactic acidosis

## Abstract

Electron transport chain (ETC) disorders are a group of rare, multisystem diseases caused by impaired oxidative phosphorylation and energy production. Deficiencies in complex III (CIII), also known as ubiquinol–cytochrome *c* reductase, are particularly rare in humans. Ubiquinol–cytochrome *c* reductase core protein 2 (*UQCRC2*) encodes a subunit of CIII that plays a crucial role in dimerization. Several pathogenic *UQCRC2* variants have been identified in patients presenting with metabolic abnormalities that include lactic acidosis, hyperammonemia, hypoglycemia, and organic aciduria. Almost all previously reported *UQCRC2*-deficient patients exhibited neurodevelopmental involvement, including developmental delays and structural brain anomalies. Here, we describe a girl who presented at 3 yr of age with lactic acidosis, hyperammonemia, and hypoglycemia but has not shown any evidence of neurodevelopmental dysfunction by age 15. Whole-exome sequencing revealed compound heterozygosity for two novel variants in *UQCRC2*: c.1189G>A; p.Gly397Arg and c.437T>C; p.Phe146Ser. Here, we discuss the patient's clinical presentation and the likely pathogenicity of these two missense variants.

## INTRODUCTION

The oxidative phosphorylation system consists of five multiprotein complexes localized to the mitochondrial inner membrane. Complexes I–IV comprise the electron transport chain (ETC) and complex V (ATP synthase) uses the proton gradient produced by the ETC to convert ADP to ATP. Defects in any of these complexes can cause human diseases manifesting as metabolic perturbations and dysfunction of many different organ systems, particularly the brain. Complex III (CIII), also known as ubiquinol–cytochrome *c* reductase, is a homodimeric complex that consists of 11 subunits per monomer. Ten of these subunits are encoded by nuclear genes and the 11th, cytochrome *b*, is encoded by mitochondrial DNA (Meunier et al. 2013). CIII deficiency is one of the rarest ETC disorders (Fernández-Vizarra and Zeviani 2015; Gaignard et al. 2017). Pathogenic variants resulting in CIII deficiency have been identified in 12 genes, including *CYTB*, *UQCRQ*, *UQCC2*, *UQCC3*, *UQCRB*, *BCS1L*, *CYC1*, *TTC19*, *LYRM7*, *UQCRFS1*, *UQCRH*, and *UQCRC2* (Fernández-Vizarra and Zeviani 2015; Burska et al. 2021; Vidali et al. 2021). Ubiquinol–cytochrome *c* reductase core protein 2 (*UQCRC2*) encodes core protein 2 of CIII. To date, several patients with pathogenic variants in *UQCRC2* have been reported. The missense variant c.547C>T; p.Arg183Trp was found in four homozygous individuals presenting with lactic acidosis and hypoglycemia at birth (Miyake et al. 2013; Gaignard et al. 2017). c.665G>C; p.Gly222Ala was found in a homozygous female presenting with lactic acidosis, normal blood glucose, and severe neurologic manifestations (Burska et al. 2021). A patient with Leigh-like syndrome was found to be a compound heterozygote for two variants: c.1340C>A:p.T447K and c.613-3->A (Ogawa et al. 2020). Six other patients with likely pathogenic *UQCRC2* variants were recently identified, emphasizing the clinical variability of defects in this subunit (Bansept et al. 2023). Here, we present the case of a now 15-yr-old girl who presented at age 3 with lethargy, vomiting, leg pain, and altered mental status. She experienced recurrent episodes of metabolic decompensation, including hypoglycemia and lactic acidosis, and was found by whole-exome sequencing (WES) to be heterozygous for two novel missense variants in *UQCRC2* (c.1189G>A; p.Gly397Arg and c.437T>C; p.Phe146Ser).

## RESULTS

### Clinical Presentation and Family History

The previously healthy female patient was born full term to a 38-yr-old mother, with no prenatal or perinatal complications. Her first newborn screen showed elevated tyrosine and long-chain acyl carnitines, but the second newborn screen was reported as normal. The patient first presented to the emergency department at age 3 with lethargy, altered mental status, and vomiting. Her parents reported that in the previous 24 h, she had refused to eat and complained of leg pain localized to her knees. She had tachycardia and hypoactive bowel sounds, but her physical examination was otherwise normal. Lab tests revealed ketonuria and hypoglycemia (blood glucose 25 mg/dL; reference range: 74–127 mg/dL); an arterial blood gas upon arrival revealed a pH of 7.19 and pCO_2_ of 20 mmHg. Other lab tests revealed a bicarbonate of 8 mEq/L (reference range 18–26 mEq/L); lactate of 7.0 mmol/L (reference range: 0.7–2.5 mmol/L); anion gap of 27 mEq/L (reference range: 7–16 mEq/L); and ammonia of 204 µM (reference range: 21–50 µM). She received a bolus of intravenous 10% dextrose for the hypoglycemia and 2 mEq/kg of bicarbonate. This improved the acid–base status somewhat (pH 7.28, pCO_2_ 30 mmHg, bicarbonate 14 mEq/L, and base deficit of 13) without changing the lactate concentration. Her white blood cell count was elevated at 36.6 × 10^9^/L (reference range: 5.0–14.5 × 10^9^/L). Blood, urine, and cerebrospinal fluid (CSF) cultures did not grow any organisms. A head computed tomography (CT) was normal. She was transferred to the pediatric intensive care unit, NPO and on D10 ½ NS for further evaluation and monitoring. The fluids were later changed to D5 ½ NS. The hyperammonemia and lactic acidosis resolved the following day, and she tolerated a full diet. She was discharged home the day after that.

The patient experienced recurrent episodes of metabolic decompensation, presenting similarly, with a frequency of 1–2 monthly episodes for almost 5 yr. The frequency then decreased to ∼3–4 episodes per year, usually triggered by acute illnesses. Consistent metabolic features during these episodes included hypoglycemia, elevated blood lactate, and metabolic acidosis with a high anion gap. Ammonia levels were routinely followed, but after her initial episode, they never exceeded 61 µM. Transaminases were also essentially normal during and between these episodes. An extensive biochemical workup revealed abnormal acylcarnitines (marked elevation of acetylcarnitine, modest elevation of hydroxybutyrylcarnitine, and minor elevations of multiple other species), elevated 3-hydroxy fatty acids, elevated pyruvate, and several urine organic acid abnormalities including massive excretion of lactate, ketones, dicarboxylic acids, and 3-hydroxydicarboxylic acids during episodes of decompensation, which normalized when she was in her healthy state. The recurrent ketosis made disorders of long- and medium-chain fatty acid oxidation unlikely, but short-chain 3-hydroxylacyl-CoA dehydrogenase deficiency (SCHAD) was considered (Popa et al. 2011). However, a SCHAD enzyme assay was normal. Amino acid screening showed elevated glutamine, alanine, tyrosine, lysine, and proline in plasma and elevated alanine, taurine, and glycine in the urine.

The patient has no other medical conditions. She uses ondansetron as needed for nausea or vomiting and dicyclomine for abdominal pain. She follows a healthy diet and does not avoid any food groups. Regarding the family history, her parents report no consanguinity. No similar episodes of hypoglycemia, acidosis, or other metabolic disorders were reported in other family members. The patient lives with four healthy full siblings and has three healthy paternal half-siblings. Her paternal grandmother had one male stillbirth of unknown cause. A paternal uncle was diagnosed with fatty liver and is on pharmacological therapy.

### Genomic Analyses

A series of targeted sequencing tests were first performed. The presence of hypoglycemia and hyperlactatemia after feeding raised the suspicion of glycogen storage disease type 0 (GSD-0), but sequencing of the glycogen synthase 2 (*GYS2*) gene, responsible for GSD-0, identified only one heterozygous, unclassified missense variant (c.1636A>G) predicted to be benign. Other considerations included fructose-bisphosphatase 1 (*FBP1*) deficiency and pyruvate carboxylase (*PC*) deficiency, but sequencing revealed no significant variants. Chromosomal microarray testing was also normal.

**Figure 1.**
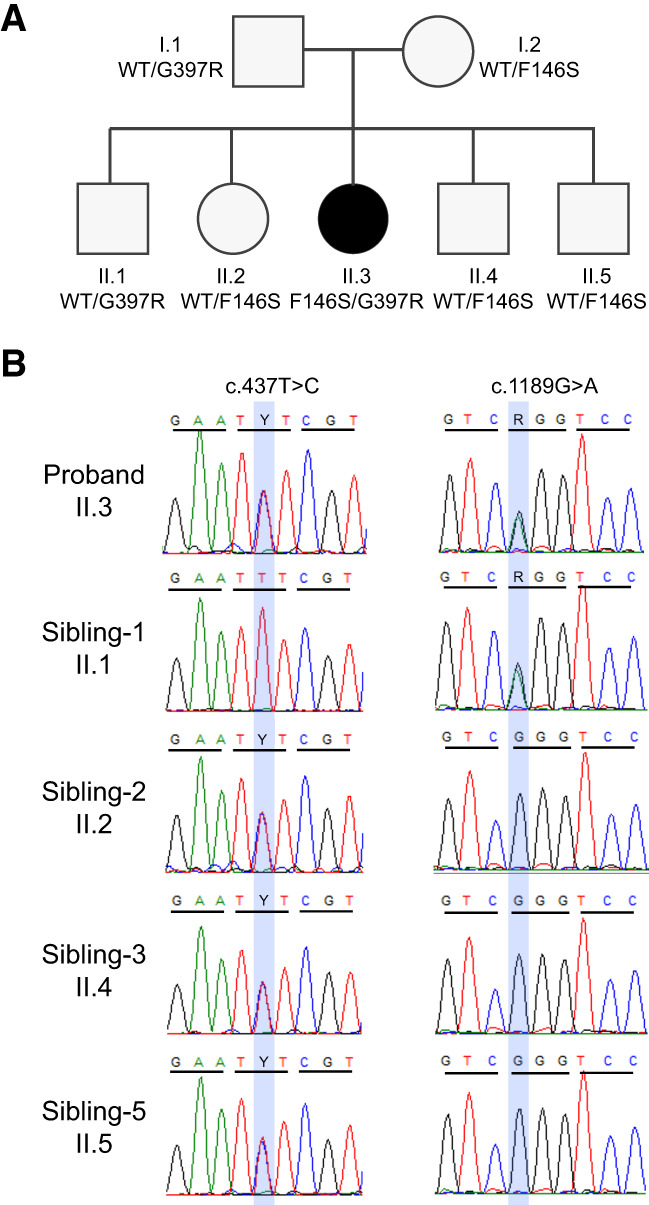
Compound heterozygosity for *UQCRC2* variants in a proband with recurrent episodes of lactic acidosis and hypoglycemia. (*A*) Pedigree and genotypes of *UQCRC2* proband (II.3), her parents, and her siblings. Each parent is heterozygous for one of the variants observed in the proband. The eldest brother is heterozygous for the G397R variant, whereas the other three siblings are heterozygous for the F146S variant. (*B*) Chromatogram of *UQCRC2* sequences from the proband (II.3) and her siblings. The relevant variants are highlighted by the blue box.

Finally, clinical WES (variants in Table 1) identified compound heterozygous variants of unknown significance in *UQCRC2*: c.1189G>A; p.Gly397Arg and c.437T>C; p.Phe146Ser, which were inherited from the father and mother, respectively (Fig. 1A). Sanger sequencing confirmed these variants in this patient, and further demonstrated that all four full siblings are carriers for one of the two variants (Fig. 1B).

**Table 1.** Variant table

Gene	Chromosome	HGVS DNA reference	HGVS protein reference	Variant type	Predicted effect	dbSNP/dbVar ID	Genotype
Ubiquinol–cytochrome *c* reductase core protein 2 (*UQCRC2*)	16p12.2 Exon 13	NM_003366.2:c.1189G>A	NP_003357.2:pGly397Arg	Substitution	PolyPhen, possibly damaging; SIFT, deleterious	rs1384782297	Compound heterozygous
Ubiquinol–cytochrome *c* reductase core protein 2 (*UQCRC2*)	16p12.2 Exon 6	NM_003366.2:c.437T>C	NP_003357.2:p.Phe146Ser	Substitution	PolyPhen, probably damaging	NA	Compound heterozygous

(HGVS) Human Genome Variation Society, (DNA) deoxyribonucleic acid, (dbSNP) Database of Single Nucleotide Polymorphisms, (dbVar ID) Database of Human Genomic Structural Variation Identification.

**Figure 2.**
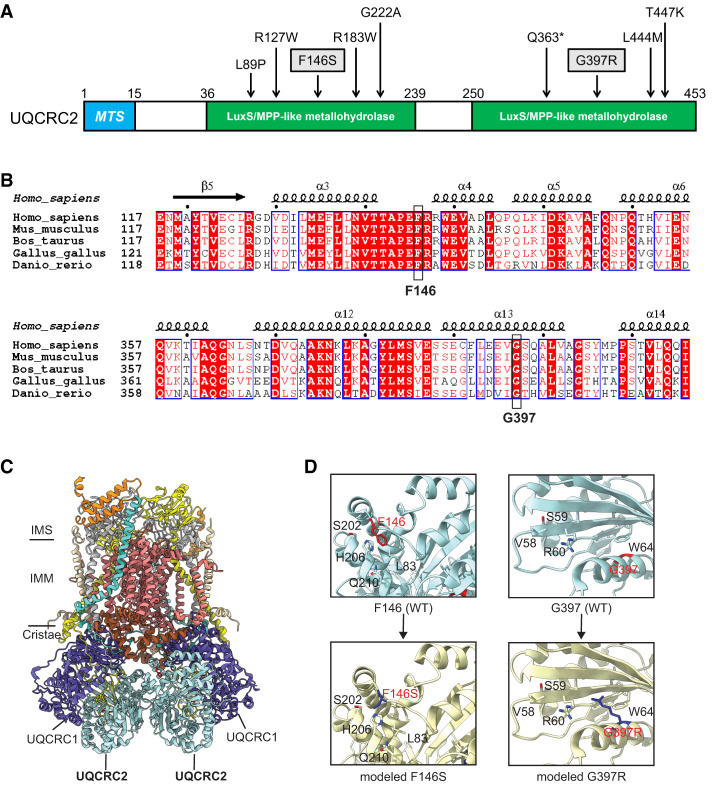
Structural representation of selected *UQCRC2* residues with pathogenic variants. (*A*) Schematic of human *UQCRC2* protein illustrating known functional domains. The variants observed in the patient described here are indicated in boxes. Other *UQCRC2* variants reported in the literature are also shown. (MTS) Mitochondrial targeting sequence, (LuxS/MPP-like metallohydrolase) *S*-ribosylhomocysteinase/mitochondrial processing peptidase-like metallohydrolase. (*B*) Structure-based alignment showing conservation of the F146 and G397 residues, shown in black boxes, across multiple species. (*C*) Three-dimensional structure of human CIII model. (*D*) The enlargements show the local interaction for each mutated residue as described in the text. (IMM) Inner mitochondrial membrane, (IMS) inner mitochondrial space.

Human UQCRC2 has two LuxS/MPP-like metallohydrolase domains. All the previously reported missense variants and the two identified in our patient are located within either of these two enzymatic domains (Fig. 2A). Structure-based alignment showed that both of the residues altered in our patient, F146 and G397, are highly conserved across multiple species (Fig. 2B). Cryogenic electron microscopy structural studies have revealed that human CIII contains two UQCRC2 subunits, each interacting with UQCRC1 on the surface of cristae facing the mitochondrial matrix (Fig. 2C). F146 is located in a loop region, and mutation of F to S might disturb its interaction with the proximal residues, including L83, S202, H206, and Q210 (Fig. 2D). Mutation of G397 to R (G397R) in an α-helix likely creates steric clashes between the arginine's side chain and residues 58–64, especially W64, within the β-sheet. These steric clashes may induce conformational changes or deformation of the three-dimensional structure of the UQCRC2, leading to impaired super complex assembly.

### Treatment Outcomes

During the episodes of metabolic decompensation, the patient received intravenous dextrose infusions to correct her hypoglycemia and fluid resuscitation for vomiting and dehydration. With this treatment, her episodes resolved quickly, usually resulting in discharge within 3 d. She has had no long-term sequelae from these events. The patient has maintained regular follow-ups in the Pediatric Genetics and Metabolism clinic every 4–6 mo. Considering the role of ubiquinone in transferring electrons from complexes I and II to complex III, supplementation with 200 mg daily of over-the-counter Coenzyme Q10 (ubiquinone) was contemplated but not initiated. At the time of this report, she is 15-yr-old, Tanner stage 4 with menarche at a normal age. She has normal intelligence and development. She has been in age-matched classes throughout her schooling.

## DISCUSSION

Diagnosing disorders of oxidative phosphorylation can be challenging because of the variable and nonspecific clinical presentations and large number of genes involved in this process. CIII deficiency is among the least common disorders in this class (Fernández-Vizarra and Zeviani 2015). Of the 11 *UQCRC2*-deficient patients in the literature, five presented as neonates (Miyake et al. 2013; Gaignard et al. 2017; Bansept et al. 2023), and the others had disease onset from 1 to 4 yr old (Burska et al. 2021; Bansept et al. 2023). Our patient came to clinical attention at age 3 and has had essentially no chronic disability. Although she has suffered many metabolic decompensations, the frequency of these episodes was mitigated relative to most patients with neonatal onset. She, therefore, appears to have a relatively mild form of CIII deficiency. It is interesting that the first documented episode did not occur until age 3, but was followed soon thereafter by many events over the next few years. It is possible that she simply happened to avoid illnesses or other stressors severe enough to induce lactic acidosis until age 3. But it is also possible that modest, self-limited episodes of hyperlactatemia were missed before her propensity for lactic acidosis was documented at age 3.

The typical clinical presentation for patients with CIII deficiency due to *UQCRC2* mutations includes lethargy, poor feeding, vomiting, neurologic manifestations, and respiratory distress. In addition to these symptoms, our patient also complained of leg pain during her initial presentation and a few subsequent episodes. Neurologic symptoms reported in the literature include Leigh-like syndrome, motor and cognitive delay, encephalomyopathy, paleocerebellar symptoms, hypotonia, developmental delay, divergent strabismus, unsteady gait, and intention tremor (Miyake et al. 2013; Gaignard et al. 2017; Burska et al. 2021; Bansept et al. 2023). Brain magnetic resonance imaging (MRI) displayed dilated ventricles, widened subarachnoid spaces, and symmetric necrotic lesions in the brainstem of one patient (Burska et al. 2021), and small parietal and temporal infarcts in another (Miyake et al. 2013). It is important to point out that all but one of the patients previously described experienced neurologic involvement at some point; the other patient had hypotonia when presenting at age 3 but developed normally and attended college (Bansept et al. 2023). Our patient never exhibited neurological involvement and is developmentally age-appropriate at 15 yr. This may indicate a higher degree of residual CIII function in the brain of our patient relative to most of the others. The case further demonstrates that normal neurological development is possible even in patients with frequent metabolic decompensations.

Regarding the biochemical presentation, similar to our patient, the patients reported in the literature had most of the following findings: lactic acidosis, hyperammonemia, hypoglycemia, elevated serum alanine and pyruvate, elevated urinary organic acids, ketones, lactic acid, and pyruvate. Our patient also had increases in multiple acylcarnitine species and 3-hydroxy fatty acids. Thus in addition to anomalies frequently observed in oxidative phosphorylation defects, patients with *UQCRC2* defects could also have some abnormalities that are less characteristic, including hypoglycemia and hyperammonemia. As reported previously (Bansept et al. 2023), our patient's metabolic episodes generally responded briskly to dextrose infusions.

According to the standards and guidelines from the American College of Medical Genetics and Genomics (Richards et al. 2015), the *UQCRC2* variants in our patient qualify as “likely pathogenic” based on the following criteria: (1) Variants are located in a critical and well-established functional domain without benign variation; (2) variants are absent/extremely low in frequency in gnomAD database (v3.1) (allele frequency of G397R is 0.00003187 and F146S is absent); (3) variants are detected in *trans* configuration; (4) missense variants are detected in a gene that has a low rate of benign missense variation and in which missense variants are a common mechanism of disease; and (5) multiple lines of computational evidence support a deleterious effect on gene or gene product (PolyPhen-2 and SIFT identified both variants as “damaging/possibly or probably damaging”). Criteria 1, 2, and 3 are considered moderate evidence of pathogenicity and criteria 4 and 5 are considered supporting evidence of pathogenicity; together these data meet the threshold for likely pathogenic variants. The structural and evolutionary analyses of UQCRC2 further support the variant's pathogenicity in the disease observed in our patient. This case highlights two novel, likely pathogenic variants in *UQCRC2* identified in a 3-yr-old patient presenting with lactic acidosis, hyperammonemia, and lethargy. The case emphasizes the possibility of normal neurodevelopment despite frequent metabolic decompensations in this disease and highlights variable severity and organ involvement in patients with CIII deficiency caused by *UQCRC2* mutations.

## METHODS

The patient and her parents were enrolled in an observational clinical research study (NCT02650622) approved and overseen by the Institutional Review Board (IRB) at UT Southwestern. Written informed consent and blood samples were obtained from the patient and her parents as part of the prospective, nonrandomized, nonblinded observational study of the Genetic and Metabolic Disease Program (study protocol STU 112014-001).

Genomic DNA from the patient and her parents was extracted from whole blood and processed for WES at the Medical Genetics laboratories/Whole Genome laboratory at Baylor College of Medicine. For massively parallel sequencing, the postcapture library DNA was subjected to sequence analysis on an Illumina HiSeq platform for 100-bp paired-end reads. The following quality control metrics of the sequencing data were generally achieved: >70% of reads aligned to target, >95% target base covered at >20×, >85% target base covered at >40×, and mean coverage of target bases >100×. This test may not provide detection of certain genes or portions of certain genes because of local sequence characteristics or the presence of closely related pseudogenes. Gross deletions or duplications or changes from repetitive sequences may not be accurately identified by this methodology. The WES test also includes a mitochondrial genome screening. The mitochondrial genome was amplified by a long-range polymerase chain reaction (PCR) and subjected to paired-end library construction and Illumina HiSeq 2000 sequencing analyses. The mean depth of coverage for targeted bases is >25,000× by this assay. Only pathogenic point changes and large rearrangements with heteroplasmy >20% (i.e., clinically relevant pathogenic changes) were reported. The c.1189G>A and c.437T>C *UQCRC2* variants were confirmed in the proband by a research-based WES analysis, which had been conducted in parallel to the clinical WES (Supplemental Table S1). To evaluate the *UQCRC2* variants in her siblings, the targeted regions (G397 and F146) were PCR-amplified from genomic DNA using the following primer pairs: For G397: forward: 5′-GATTGCACAACCGGATCCAG-3′, reverse: 5′-ACTGCCCAAGGACGCTTAT-3′. For F146: forward: 5′-TTCAGTGTGACCGCAACAAG-3′, reverse: 5′-GAATTGCTTGAACCCGGGAG-3′. The patient's and sibling's genotypes were then determined by Sanger sequencing (Genewiz). Chromosomal microarray (CMA) was performed by the Medical Genetics laboratories at Baylor College of Medicine. CMA by the Affymetrix Cytoscan HD is a molecular cytogenetic test which contains 2.6 million markers (around 750,000 SNP probes and 1,900,000 nonpolymorphic oligonucleotide probes) and has an average resolution of 1 probe/3 kb in targeted regions and 1 probe/5 kb in the backbone. It is designed to detect losses and gains representing deletions or duplications for a wide array of clinically significant regions of the human genome. This test is designed to detect copy-number changes >300 kb and uniparental isodisomies/absence of heterozygosity (AOH). AOH < 5 Mb in size was not reported. The detection rate of heterodisomies is currently not known for this assay. This assay also does not detect balanced translocations, inversions, or low-level mosaicism. The SCHAD enzyme assay was performed at the Children's Hospital of Philadelphia. FBP1 gene sequencing for fructose-1,6-bisphosphate deficiency was performed at Prevention Genetics. Using the patient's genomic DNA, they amplified and sequenced the full coding regions of the indicated exons as well as around 50 bases of flanking noncoding sequences. They then aligned and compared the patient's sequences with the reference sequences. All differences from the reference sequences (sequence variants) were reported. Nomenclature for sequence variants was taken from the Human Genome Variation Society recommendations. As required, DNA was extracted from the patient using a 5 Prime ArchivePure DNA Blood Kit. PCR was used to amplify the indicated exons plus additional flanking intronic or other noncoding sequence. Products were resolved by electrophoresis on an ABI 3730 × 1 capillary sequencer. Sequencing was performed separately in both the forward and reverse directions. *PC* sequence analysis for pyruvate carboxylase deficiency was performed by the Medical Genetics laboratories at Baylor College of Medicine. The coding regions of the *PC* (NM_00920.3) gene are PCR-amplified and then sequenced in the forward and reverse directions using automated fluorescent sequencing methods. Nucleotide 1 corresponds to the A of the start codon ATG. Variants detected in exons and in introns within up to 20 bp of the exon/intron boundaries are reported. This analysis does not detect large heterozygous deletions or duplications, inversions, or mutations with promoter or deep intronic regions. *GYS2* sequencing for GSD-0 was also performed by Medical Genetics laboratories at Baylor College of Medicine. Coding exons and immediate flanking intronic regions of the *GYS2* gene located at 12p12.1 are PCR-amplified and then sequenced in the forward and reverse directions using automated fluorescent dideoxy sequencing methods. GenBank (NCBI) ID NM_021957.3 is used as the reference sequence.

## ADDITIONAL INFORMATION

### Data Deposition and Access

The two new *UQCRC2* variants were submitted to ClinVar (https://www.ncbi.nlm.nih.gov/clinvar/) and can be found under accession numbers VCV002446878 and VCV002446877. Patient consent was not obtained for deposition of raw sequencing data.

### Ethics Statement

The patient, her parents, and four siblings were enrolled in a study focused on developmental and metabolic anomalies entitled “Genetic Regulators of Metabolism and Development in Children,” approved by the IRB at the University of Texas Southwestern Medical Center (UTSW).

### Informed Consent

Written informed consent was obtained from the parents as the patient and her siblings were minors at the time of recruitment. All procedures were in accordance with the ethical standards of the responsible committee on human experimentation (institutional and national) and with the Helsinki Declaration of 1975, as revised in 2000 (5). Informed written consent was obtained from the patient and her parents for being included in the study.

### Acknowledgments

We thank the patients who participated in this study. R.J.D. is funded by the Howard Hughes Medical Institute and the Robert L. Moody, Sr. Faculty Scholar endowment. We thank the Baldridge family for their support of the CRI Metabolomics Facility. We thank the University of Texas Southwestern Medical Center Bioinformatics Core Facility, funded by the Cancer Prevention and Research Institute of Texas (CPRIT, RP150596), for the genomics analysis pipeline used in this study.

### Author Contributions

L.A.H. and R.C.H. reviewed the literature. L.A.H., R.C.H., and P.P. collected the patient data by chart reviews. All authors wrote and edited the manuscript and analyzed and interpreted the research data. R.J.D. and G.K.G. obtained and interpreted clinical data, provided direct patient care, and provided clinical expertise on complex III deficiency. All authors have approved the current version of the manuscript and its submission to *CSH Molecular Case Studies*.

### Funding

This work was funded by the Howard Hughes Medical Institute and the Robert L. Moody, Sr. Faculty Scholar endowment.

### Competing Interest Statement

R.J.D. is a founder and advisor for Atavistik Bioscience. He is also a member of the Scientific Advisory Board for Agios Pharmaceuticals and Vida Ventures.

### Referees

Jaya Ganesh

Anonymous
